# Round the Hospitals

**Published:** 1921-08-06

**Authors:** 


### ROUND THE HOSPITALS.

The visit of the Prince of Wales to the Royal Victoria Hospital at Folkestone on July 28 was a memorable event in the history of the institution. In itself the occasion was highly interesting, for the opening of the long-needed nurses' home marks a definite advance in the prosperity and attractions of the nurse-training school. And the Prince has the inherited gift of lifting ordinary functions into an atmosphere of distinction. None who were present will ever forget his earnest words of congratulation to the doctors and nurses on their record of work in peace and in war, recalling particular instances in which exceptional demands had been magnificently met by the hospital staff. Among those with whom the Prince conversed was Mrs. Bowbrick, who was able to be wheeled in an invalid-chair, having been an inmate of the hospital since she was injured in the air-raid on Folkestone in 1917.

Hard on the opening of the English Register comes the official announcement of the opening of the Scottish Register. The delay in this has been caused by the contention of the Scottish Board of

**ROUND THE HOSPITALS**—(*continued*).

Health that nurses on their Register of certificated fever nurses should be placed on the Council's Register of existing general nurses. The Scottish Board of Health has now withdrawn this contention, and it has approved the Council's rules. Applications for registration from all classes of nurses who have either been trained in Scotland or are resident or practising there can therefore now be received. The first step is to apply to the Registrar, General Nursing Council for Scotland, 13 Melville Street, Edinburgh, for forms of application for registration. Applicants should state the part or parts of the Register to which they wish to be admitted, viz.: (1) General (for females only); and the following supplementary Registers: (2) Male; (3) Mental; (4) Mental Defectives; (5) Sick Children; (6) Infectious Diseases.

---

A DELIGHTFUL garden-party was given at the Nurses' Home by the Matron, Miss Baillie, and the nursing staff of the Bristol Royal Infirmary, on July 21. The Duchess of Beaufort opened the proceedings and a large company of friends of the Infirmary gathered to do honour to the occasion. The object of the fête was to raise funds for various purposes conducive to the nurses' comfort and well-being in off-duty times, and some very well-furnished flower and fancy stalls brought in a good return. Alderman Sheppard, Chairman of the Committee, in welcoming the Duchess, claimed that nursing was the most honourable profession women can undertake, and alluded with pride to the fact that the Bristol Royal Infirmary was one of the first outside London to establish a preliminary training centre for probationers. The Duchess, who was presented with a doll dressed as a nurse, in lieu of a bouquet, spoke with great warmth of the help given to her war hospital at Badminton by Miss Baillie, who practically superintended it, and of the debt she and her family owed to nurses of the Royal Infirmary, who had often nursed them in illness.

---

THE Birmingham District Nursing Society is curiously unsuccessful in eliciting support in that rich and generous city for the care of the sick poor. The Lady Mayoress has now made an urgent appeal for nursing appliances, and an appeal has also been made by the Chairmen of the Central and South Homes House Committee for the ordinary requirements of the nurses in their every-day visits, without which they are hampered at every turn. When we call to mind the splendid response made to the efforts of the College of Nursing at the recent fair we are tempted to advise the Committee to call some of the organisers into counsel. The district nurses need an intelligently organised publicity department working on their behalf. With Birmingham people to know the need is to meet it.

---

DERBY has come very near to the development in all its branches of a comprehensive nursing service. The Royal Derby and Derbyshire Nursing and Sanitary Association, which has recently held its annual meeting, presided over by the Mayor, employs in all seventy-six nurses, forty-one on the private staff, twenty in the Nightingale Home, District, and Home of Rest, six in outlying districts. Besides this there are ten probationers in training, and many pupils training in midwifery and general nursing. During the year sixty-one pupils passed the C.M.B. Examination, and forty completed twelve months' training in midwifery and general district work. To meet the increasing demand for beds at the Nightingale Home, the Building Committee has decided to purchase Park House, Duffield Road, for the sum of £5,000 for the use of private maternity patients. Sister Hodgkinson, the superintendent of the district nurses, has completed twenty years' service, and was warmly congratulated on the efficiency of her staff.

---

WE rejoice to learn that since Saturday last a decided improvement has manifested itself in Miss Cox-Davies' condition, and that this improvement has been steadily maintained. There is, we learn, good ground for hope that it will continue.

---

SCHOLARSHIPS are in the air. Last week we announced the results of the College and Cowdray sister-tutor studentships, and this week we are able to give the results of the scholarships and examination records for 1921 of the Nightingale Training School at St. Thomas's Hospital. Three scholarships have been awarded tenable at King's College for Women, the winners being Miss Ella M. Thompson, Miss Lucy Duff-Grant, and Miss May Wynne. They are, of course, all graduates of the Nightingale School; the two latter are of recent certification, Miss Duff-Grant's subsequent work being that of temporary night superintendent at St. Thomas's Hospital and holiday sister's duties there. Miss Wynne has also done holiday sister's duties. Miss Thompson gained her certificate in 1911, and was subsequently sister at the Rotunda Hospital, Dublin, and night sister, home sister and lecturer at the Middlesex Hospital; in 1917 she joined the Colonial Nursing Service, and was assistant lady superintendent and acting lady superintendent of the King George Hospital, Lucknow, from 1918 to 1920. At present she is taking holiday sister's duties at the Middlesex Hospital. Another scholarship winner is Miss Margaret A. James, to whom has been granted the scholarship awarded by the British Red Cross Society in connection with the scheme of training for international students. It is tenable at Bedford College and is for public-health work. Miss James holds the certificate of the Nightingale Training School and passed class "A" in all examinations.

---

THE Nightingale Medals of St. Thomas's Hospital, 1921, have been awarded to Nurse Eleanor L. Morris (gold medal), Nurse Sydney Jackson (silver medal, qualified for the gold medal), and Nurse Alice M. Harvey (bronze medal, qualified for the gold medal). Nurses Margaret Philpott, Mary B. Higgens, and Mary V. McIlroy also qualified for the gold medal.

